# A pilot study of basal ganglia and thalamus structure by high dimensional mapping in children with Tourette syndrome

**DOI:** 10.12688/f1000research.2-207.v1

**Published:** 2013-10-08

**Authors:** Alton C. Williams, Marie E. McNeely, Deanna J. Greene, Jessica A. Church, Stacie L. Warren, Johanna M. Hartlein, Bradley L. Schlaggar, Kevin J. Black, Lei Wang

**Affiliations:** 1 Washington University School of Medicine, St. Louis, MO 63110, USA; 2 Division of Biology and Biomedical Sciences, Washington University, St. Louis, MO 63110, USA; 3 Current affiliation: Centene Corporation, St. Louis, MO 63105, USA; 4 Department of Psychiatry, Washington University School of Medicine, St. Louis, MO 63110, USA; 5 Department of Radiology, Washington University School of Medicine, St. Louis, MO 63110, USA; 6 Department of Neurology, Washington University School of Medicine, St. Louis, MO 63110, USA; 7 Current affiliation: Department of Psychology in The College of Liberal, University of Texas- Austin, Austin, TX 78712, USA; 8 Current affiliation: Department of Mental Health, St. Louis VA Medical Center, St. Louis, MO 63110, USA; 9 Department of Anatomy & Neurobiology, Washington University School of Medicine, St. Louis, MO 63110, USA; 10 Department of Pediatrics, Washington University School of Medicine, St. Louis, MO 63110, USA; 11 Current affiliation: Department of Psychiatry and Behavioral Sciences, Northwestern University Feinberg School of Medicine Chicago, Chicago, IL 60611, USA; 12 Current affiliation: Department of Radiology, Northwestern University Feinberg School of Medicine Chicago, Chicago, IL 60611, USA

## Abstract

***Background*: **Prior brain imaging and autopsy studies have suggested ****that structural abnormalities of the basal ganglia (BG) nuclei may be present in Tourette Syndrome (TS). These studies have focused mainly on the volume differences of the BG structures and not their anatomical shapes.  Shape differences of various brain structures have been demonstrated in other neuropsychiatric disorders using large-deformation, high dimensional brain mapping (HDBM-LD).  A previous study of a small sample of adult TS patients demonstrated the validity of the method, but did not find significant differences compared to controls. Since TS usually begins in childhood and adult studies may show structure differences due to adaptations, we hypothesized that differences in BG and thalamus structure geometry and volume due to etiological changes in TS might be better characterized in children.

***Objective*: **Pilot the HDBM-LD method in children and estimate effect sizes.

***Methods*:** In this pilot study, T1-weighted MRIs were collected in 13 children with TS and 16 healthy, tic-free, control children. The groups were well matched for age.  The primary outcome measures were the first 10 eigenvectors which are derived using HDBM-LD methods and represent the majority of the geometric shape of each structure, and the volumes of each structure adjusted for whole brain volume. We also compared hemispheric right/left asymmetry and estimated effect sizes for both volume and shape differences between groups.

***Results*:** We found no statistically significant differences between the TS subjects and controls in volume, shape, or right/left asymmetry.  Effect sizes were greater for shape analysis than for volume.

***Conclusion*:** This study represents one of the first efforts to study the shape as opposed to the volume of the BG in TS, but power was limited by sample size. Shape analysis by the HDBM-LD method may prove more sensitive to group differences.

## Introduction

Tourette syndrome (TS) is a chronic idiopathic syndrome characterized by the appearance of both vocal and motor tics during childhood or adolescence ^1, 2^. Tics are repetitive, stereotyped, suppressible movements or vocalizations that may include blinking, abdominal tensing, sniffing, or throat clearing ^3^. TS affects approximately 0.5% of school-age children, but its causes and pathophysiology are not yet well understood ^4^.

It has been suggested that problems with activity modulation in the basal ganglia and thalamus may contribute to the inability of TS patients to exercise behavioral inhibition ^5, 6^ as a result of these structures’ effects on behavioral inhibition via the prefrontal, parietal, temporal, and cingulate cortices ^7^. The basal ganglia and thalamus modulate cortical activity through cortico-basal ganglia-thalamo-cortical loops, composed of connections from the frontal cortex to the striatum, the striatum to the globus pallidus, substantia nigra, and thalamus, and the thalamus back to the cortex ^8^.

Several lines of evidence support the presence of structural abnormalities in basal ganglia nuclei in individuals with TS ^4^. Autopsy studies have found abnormalities within the basal ganglia, including increased number of neurons in the globus pallidus interna, decreased density and number of neurons in the globus pallidus pars externa, and decreased parvalbumin and choline acetyltransferase staining cholinergic interneurons in the caudate nucleus and putamen ^9, 10^. However, since TS is rarely a fatal disease, the number of autopsied cases is limited ^11^. Case studies of focal brain lesions have demonstrated new tic onset after lesions to the prefrontal cortex, thalamus, and basal ganglia ^12^. In addition, encephalitis lethargica, frontal lobe degeneration, Huntington disease, Wilson disease, and other degenerative illnesses are associated with tics ^12^. Further, some TS patients have benefitted from deep brain stimulation of the globus pallidus and thalamus in TS ^13– 16^. Collectively, these observations suggest a role for the basal ganglia, thalamus, and frontal cortex in tics.

Neuroimaging studies can be especially beneficial for studying structural abnormalities because they allow longitudinal study design, reduced investigator and sampling bias, and are relatively non-invasive. A number of MRI studies have examined anatomical volumes and cortical thickness in children and adults with TS and reported significant differences in various brain regions, including the caudate, sensorimotor and prefrontal cortex, and corpus callosum ^17^. Most consistently, basal ganglia volumes were found to be smaller in TS subjects compared with healthy controls, but neuroanatomical shape differences and asymmetry abnormalities have not yet been consistently described ^18– 24^.

Large-deformation high dimensional brain mapping (HDBM-LD) is a computational anatomy tool that reduces the potential for human error in image analysis by further automating elements of image analysis. It has been successfully employed in characterizing shape and volume abnormalities of the hippocampus in neuropsychiatric disorders such as schizophrenia ^25– 27^, dementia of the Alzheimer type ^28– 31^, depression ^32^ and epilepsy ^33^. It has also been applied to examine the thalamus in schizophrenia ^34^.

HDBM-LD was applied to assess volume and shape differences in putamen, caudate nucleus, nucleus accumbens, globus pallidus, and thalamus in 15 adults with TS and 15 matched controls. No differences in volume or shape were found ^35^. However, TS begins before adulthood. Several structural imaging studies in TS have found an interaction between regional brain volumes and age ^21, 22^. It has been suggested that differences seen in adult studies may reflect adaptations or selection bias rather than changes etiologically relevant to TS ^20^. Thus the present study applied HDBM-LD to investigate the volume and shape of these structures in children. We hypothesized that we would find reduced volume, abnormal shape, or abnormal right-to-left asymmetry in one or more of these structures, compared to age-matched controls. Given that there were no prior studies using the HDBM-LD method to analyze brain structures of children with TS in the literature, another goal of this pilot study was to estimate the effect size of these measures in this population.

## Materials and methods

### Ethics statement

A parent of each subject gave written informed consent to participate in the study, and each subject assented to participation. The study was approved by the Washington University Human Studies Committee (approval # 03-1282).

### Participants

This study included 13 children with TS (mean age (SD) = 12.44 (2.22), 3 female, 12 right-handed) and 16 healthy controls (mean age (SD) = 12.39 (1.92), 2 female, 15 right-handed). A movement disorders-trained physician examined all TS subjects and 10 of the control subjects. The remaining control subjects underwent neuropsychological evaluation as described previously ^36^. Exclusion criteria were: inability to give informed consent, contraindication to MRI, currently symptomatic major depression, or lifetime history of mental retardation, autism, psychosis, mania, anorexia, bulimia, or drug abuse. All TS subjects met DSM-IV-TR criteria either for Tourette’s Disorder or Chronic Tic Disorder. Disease duration and severity and other clinical characteristics are summarized in Table 1.

**Table 1.** Subject characteristics.

	TS group	Control group
*n*	13	16
Age at scan (mean ± sd)	12.44 ± 2.22	12.39 ± 1.92
Sex	3F/10M	2F/14M
Handedness	12R/1A	15R/1L
Years since onset of tics ± sd	4.31 ± 2.69	NA
YGTSS total tic score* ± sd	19.00 ± 11.66	NA
Number with ADHD diagnosis	4	0
Number with OCD diagnosis	5	0
Number who reported currently taking medication:
Atypical neuroleptics	1	0
Typical neuroleptics	1	0
Stimulants	1	0
Benzodiazepines	0	0
Selective serotonin reuptake inhibitors	3	0
alpha-2 agonists	5	0
Tricyclic antidepressants	2	0
Tetracyclic antidepressants	1	0
1st generation antihistamines	2	0
Number who reported past use of medication:
Atypical neuroleptics	1	0
Typical neuroleptics	1	0
Stimulants	3	0
Benzodiazepines	1	0
Selective serotonin reuptake inhibitors	1	0
alpha-2 agonists	5	0
Tricyclic antidepressants	0	0
Tetracyclic antidepressants	0	0

*YGTSS total tic score includes only the motor tic and vocal tic subscores for a maximum of 50 points. R = right-handed, L = left-handed, A = ambidextrous.

### Image acquisition and preprocessing

A 1.5 T Siemens Vision system with a standard head receiver coil was used to collect T1-weighted MR structural images. Prior to scanning sessions, the transmitter was tuned and the main field was shimmed. Anatomic images used a 3D T1-weighted sequences (MPRAGE, 1x1x1.25 mm ^3^ voxels) ^37^. Individual MPRAGE collections lasted approximately 6.5 minutes.

Initial image processing was done as described previously ^35, 38^. Using Analyze ^TM^ software (Rochester, Minnesota), images were linearly rescaled so that voxels with intensity two standard deviations above the mean in the corpus callosum were mapped to 255, and voxels with intensity levels two standard deviations below the mean in the lateral ventricles were mapped to 0.

Whole-brain volume for each subject, excluding the ventricles, was obtained from FreeSurfer ( http://surfer.nmr.mgh.harvard.edu/) ^39^.

### Large-Deformation High-Dimensional Brain Mapping (HDBM-LD)

HDBM-LD was used to determine the volume and shape of the brain structures of interest in all subject scans, as described in detail elsewhere ^35^. Briefly, on each subject’s brain image, a single rater (MEMcN) marked 27 points on the boundaries of the basal ganglia and thalamus in each hemisphere, which were used as an initial step to roughly align the brain image to a labeled standard brain image (template). From this starting point a differentiable, invertible transformation was computed that mapped all voxels of the subject’s image to the template. Using this transformation, the labels on the template image are automatically assigned to the corresponding voxels of each subject’s image. The authors checked the segmentation of each subject’s MR image by visual inspection. This method is extremely reliable and has been validated against expert manual tracings ^35^.

### Brain structure volume and shape analysis

All brain structure volume and shape analysis methods were conducted as described previously ^35^. We examined five structures: caudate nucleus, nucleus accumbens, globus pallidus, putamen, and thalamus. Volume for each structure was analyzed using a repeated measures ANCOVA, with diagnostic group as the between-subjects factor, brain hemisphere as the within-subjects factor, and age and whole brain volume as covariates. The degree of volumetric asymmetry was examined with the hemisphere effect, and group differences in volumetric asymmetry were assessed by examining the group-by-hemisphere interactions. We also analyzed the total (left and right hemisphere) structure volumes using an ANCOVA. The volume ANCOVAs were repeated with other covariates and factors, including estimated total intracranial volume, sex and handedness, none of which substantively changed the results.

Brain structure shapes were determined from the inter-subject deformation vector fields provided by the HDBM-LD transformations. Eigenvalues and a complete orthonormal set of eigenvectors representing shape variation were obtained using singular value decomposition (SVD) of the pooled covariance in the population studied. The coefficients (eigenscores) associated with the eigenvalues and eigenvectors were calculated for each subject and for each structure in each hemisphere ^35, 40^. We used the eigenscores based on the first ten eigenvectors for each structure in each hemisphere in a multivariate ANCOVA to test for group differences in shape. These first ten eigenscores explained 81–92% of the total variance for each structure.

Data file (subject characteristics, volume and shape) In this data file the columns from left to right represent Diagnosis (dx=diagnosis, T = TS or chronic tic disorder, C = Control), Sex (m = male, f = female), Age at scan (in years), and handedness (L = Left, R = Right, A = Ambidextrous). Then the shape principal component (eigenvector) values are represented as demonstrated by the following example: “NaLpc01” = nucleus accumbens (Na) Left (L) principal component (pc) 01. (“Na” = nucleus accumbens, “Cd” = caudate, “Pl” = globus pallidus, “Pu” = putamen, “Th” = thalamus). Next the volumes are listed using similar notation, e.g. “volCdL” = volume in mm3 (vol) Caudate (Cd) Left (L). Finally, Freesurfer analysis values are listed using their standard notation, which can be found in the table at: http://ftp.nmr.mgh.harvard.edu/fswiki/FsTutorial/AnatomicalROI#aseg.stats - See more at: http://62.231.116.94/black/#sthash.OO28vyHo.dpuf

## Results

### Volume

Repeated-measures ANCOVAs showed no significant group effect for any structure. Structural volumes and ANCOVA statistics are shown in Table 2. Additionally, no significant hemisphere effects or group by hemisphere interactions were seen for any of the five structures examined (see Table 2).

**Table 2.** Volumes of the structures of interest (mm ^3^).

		TS (n = 13)		Control (n = 16)		ANCOVA statistics (hemisphere by dx)		
		Mean (std)	[95% CIs] mm ^3^	Mean (std)	[95% CIs] mm ^3^	F	*df*	P
Caudate	L R T	3736 (271) 3712 (545) 7448 (731)	[3581, 3890] [3401, 4023] [7030, 7865]	3667 (270) 3678 (543) 7345 (729)	[3528, 3806] [3398, 3957] [6969, 7720]	0.040	1,25	0.84
Nucleus accumbens	L R T	460 (46) 455 (50) 915 (74)	[434, 487] [426, 483] [873, 957]	462 (46) 456 (50) 918 (73)	[438, 485] [430, 481] [880, 955]	0.000	1,25	0.996
Globus pallidus	L R T	1826 (126) 1859 (145) 3685 (248)	[1754, 1898] [1776, 1942] [3544, 3827]	1804 (125) 1800 (144) 3603 (247)	[1739, 1868] [1726, 1874] [3476, 3730]	0.768	1,25	0.39
Putamen	L R T	5925 (367) 5822 (401) 11748 (724)	[5716, 6135] [5593, 6052] [11334, 12161]	5705 (365) 5671 (399) 11376 (721)	[5517, 5893] [5465, 5877] [11005, 11748]	0.487	1,25	0.49
Thalamus	L R T	8076 (557) 8143 (480) 16219 (805)	[7757, 8394] [7869, 8418] [15759, 16679]	7931(555) 7888 (478) 15819 (802)	[7645, 8217] [7642, 8134] [15406, 16232]	0.196	1,25	0.66

L = left, R = right, T = total volume. Repeated-measures ANOVA of each structure showed no significant group effect. Further, we found no hemisphere effect or group by hemisphere interactions for any of the structures (age and whole brain volume w/out ventricles as covariates).

### Shape

MANCOVAs (using the first ten eigenscores as dependent variables) for each structure in each hemisphere showed no significant group effect (see Table 3). Effect sizes (Cohen’s ƒ ^2^) for both volume and shape are provided in Table 4; the effect sizes for the shape comparisons were larger than those for the volume comparisons.

**Table 3.** Shape comparison of the thalamus and basal ganglia structures (TS vs. control).

		MANCOVA statistics		
Structure		F	*df*	P
Nucleus accumbens	L R	1.63 1.91	10,17 10,17	0.18 0.11
Caudate	L R	1.31 .739	10,17 10,17	0.30 0.68
Globus pallidus	L R	.231 .848	10,17 10,17	0.99 0.59
Putamen	L R	.285 .740	10,17 10,17	0.98 0.68
Thalamus	L R	.705 .893	10,17 10,17	0.71 0.56

L = left, R = right, T = total volume. Multivariate analysis of the first 10 eigenvectors of each structure showed no significant group effect (age as covariate).

**Table 4.** Effect sizes.

	Partial η	Cohen’s ƒ ^2^
Volumes (total structure volumes)		
Caudate	5.51 × 10 ^-3^	5.54 × 10 ^-3^
Nucleus accumbens	2.77 × 10 ^-4^	2.78 × 10 ^-4^
Globus pallidus	2.94 × 10 ^-2^	3.03 × 10 ^-2^
Putamen	6.79 × 10 ^-2^	7.29 × 10 ^-2^
Thalamus	6.42 × 10 ^-2^	6.86 × 10 ^-2^
Volumes (hemisphere * dx effects)		
Caudate	2.00 × 10 ^-3^	2.00 × 10 ^-3^
Nucleus accumbens	1.22 × 10 ^-6^	1.22 × 10 ^-6^
Globus pallidus	3.00 × 10 ^-2^	3.09 × 10 ^-2^
Putamen	1.90 × 10 ^-2^	1.94 × 10 ^-2^
Thalamus	8.00 × 10 ^-3^	8.06 × 10 ^-3^
Shapes (principal components):		
Left		
Caudate	0.436	0.773
Nucleus accumbens	0.490	0.961
Globus pallidus	0.120	0.136
Putamen	0.144	0.168
Thalamus	0.293	0.414
Right		
Caudate	0.303	0.435
Nucleus accumbens	0.530	1.128
Globus pallidus	0.333	0.499
Putamen	0.303	0.435
Thalamus	0.344	0.524

## Discussion

Using HDBM-LD, a validated method for automatic, high-dimensional mapping of basal ganglia and thalamic structures, we found no significant differences in basal ganglia volumes or shape between children with TS and matched control children. For most basal ganglia regions, this reflects the conclusions of a recent review ^17^. For instance, two groups found increased putamen volume in TS ^41, 42^, but a larger study found decreased volume ^43^. However, the majority of these studies found no abnormality in putamen, similar to the current study. Three other studies, including the HDBM-LD study in adults with TS, found no volumetric change in any basal ganglia structure ^35, 44– 46^. Possibly there is no true difference in these structures in TS when groups are matched carefully for age, sex and handedness. Alternatively, structural abnormalities in TS may be limited to certain subgroups, such as those with more severe tics or with ADHD.

On the other hand, the largest published MRI study of basal ganglia volume compared 154 adults and children with TS to 130 tic-free control subjects, and found that the caudate was 4.9% smaller in the TS group (p<0.01) ^43^. Two other groups also found lower caudate volume in samples of 18–23 TS subjects and a similar number of controls ^23, 24, 47, 48^. The possible etiologic relevance of this finding is highlighted by the observation that a smaller caudate nucleus in adolescents with TS predicts more severe symptoms in early adulthood ^49^. The largest of the studies that did not find significant decreases in caudate volume was that of Roessner *et al.* ^42^, which compared 55 subjects with TS to 42 control subjects. The other studies with negative findings regarding caudate volume, including the present one, had fewer than 20 TS subjects each. It is possible these negative results represent a Type II error.

The present study and the HDBM-LD study in adults represent some of the first efforts to study the shape (as opposed to the volume) of basal ganglia nuclei in TS, and provide effect size estimates for planning a study with larger samples.
