# Supplementary material for: A pilot study of basal ganglia and thalamus structure by high dimensional mapping in children with Tourette syndrome
Source: F1000Res. 2013 Oct 8;2:207. [Version 1] doi: 10.12688/f1000research.2-207.v1 (PMC3976104; doi:10.12688/f1000research.2-207.v1)
Supplement: Peer review history

# Peer review: Referee response for version 1

**Published:** 2014-03-27

**Authors:** Kirsten R Müller-Vahl

**Affiliations:** 1 Clinic of Psychiatry, Social psychiatry and Psychotherapy, Hannover Medical School, Hannover, Germany

In this study a relatively new and sophisticated method (using large-deformation, high dimensional brain mapping (HDBM-LD)) has been used to investigate for the first time structure geometry and volume of basal ganglia and thalamus in 13 children with Tourette syndrome (TS) compared to 16 healthy controls. Comparable to a prior study in adults with TS performed by the same group, no significant differences could be detected in volume, shape, or right/left asymmetry.

 

These findings are in contrast to several other studies suggesting a reduction of the volume of the caudate in both children and adults with TS. The major limitation of this study is the small sample size and the heterogeneity of the patients’ group with respect to comorbidities and medication. In particular, medication with neuroleptic drugs might have influenced the results, since there is evidence that exposure to antipsychotic medication may cause basal ganglia enlargement. However, the study has also several strengths including the well experienced team of researchers, low age range and inclusion of children only, and excellent matching for age, sex, and handedness. I enjoyed reading the discussion, because results obtained in children were not mixed with those in adults.

 

The introduction would benefit from a more precise clinical description. For example: tics are no longer characterized as “stereotyped” movements, abdominal tension is an untypical example for a motor tic, and tics “may” be associated with other disorders such as Wilson’s and Huntington’s disease. The title and abstract are appropriate.

 

Although this study contributes to our understanding of the neurobiology of TS, comparable to most MRI studies in TS, it is limited by the small sample size and possible influences of medication and comorbidities. I would love to read the results of a follow-up study including a larger number of patients soon.

I have read this submission. I believe that I have an appropriate level of expertise to confirm that it is of an acceptable scientific standard.

# Response

**Published:** 2014-03-27

**Authors:** Kevin J. Black

**Affiliations:** Washington University in St. Louis, USA

We appreciate Dr. Müller-Vahl's thoughtful comments.

# Peer review: Referee response for version 1

**Published:** 2014-01-24

**Authors:** Jeremy Stern

**Affiliations:** 1 Department of Neurology, St. George's Hospital and Medical School, London, UK

This study continues the quest for subtle structural abnormalities in striato-thalamo-cortical circuitry in Tourette syndrome. The technique is innovative and in this paper is negative for a relatively small number of children as it has been for a similar number of adults in the past. The technique has yielded positive results in certain other conditions. The current results are not consistent with some other larger studies of caudate volumes and the reasons for this are explored and are a common issue in this area. Given the small number of subjects with heterogeneity of comorbidities and medication history the negative result here cannot be definitive, as suggested in the discussion.

A common group of core authors in a small number of sites published the previous HDBM-LD work and so external replication, particularly of the so far positive results would be valuable. The methadology is described more fully in an earlier paper, but it is hard to confirm if it could be reproduced directly from this alone.

The title and abstract are appropriate.

I have read this submission. I believe that I have an appropriate level of expertise to confirm that it is of an acceptable scientific standard.

# Response

**Published:** 2014-02-11

**Authors:** Kevin J. Black

**Affiliations:** Washington University in St. Louis, USA

We greatly appreciate Prof. Stern's thoughtful comments about the relevance and limitations of this pilot study. The prior report (ref. 35 above) does describe the methods in detail.
